# Progress in Personalizing Chemotherapy for Bladder Cancer

**DOI:** 10.1155/2012/364919

**Published:** 2012-02-13

**Authors:** James S. Chang, Primo N. Lara, Chong-Xian Pan

**Affiliations:** ^1^Department of Internal Medicine, Division of Hematology/Oncology, University of California Davis, Sacramento, CA 95817, USA; ^2^Department of Urology, University of California Davis, Sacramento, CA 95817, USA; ^3^Department of Medicine, VA Northern California Health Care System, Mather, CA 95655, USA; ^4^UC Davis Urothelial Carcinoma Initiative, University of California Davis Cancer Center, 4501 X Street, Room 3016, Sacramento, CA 95817, USA

## Abstract

Platinum-based chemotherapy is commonly used for the treatment of locally advanced and metastatic bladder cancer. However, there are currently no methods to predict chemotherapy response in this disease setting. A better understanding of the biology of bladder cancer has led to developments of molecular biomarkers that may help guide clinical decision making. These biomarkers, while promising, have not yet been validated in prospective trials and are not ready for clinical applications. As alkylating agents, platinum drugs kill cancer cells mainly through induction of DNA damage. A microdosing approach is currently being tested to determine if chemoresistance can be identified by measuring platinum-induced DNA damage using highly sensitive accelerator mass spectrometry technology. The hope is that these emerging strategies will help pave the road towards personalized therapy in advanced bladder cancer.

## 1. Introduction

Bladder urothelial cancer is the 4th most common cancer in males and 9th in females and a major cause of morbidity and mortality worldwide. In the United States, approximately 70,530 individuals were diagnosed with bladder cancer in 2010 and 14,680 died from it [1]. Most bladder cancers in the developed world are of urothelial origin (transitional cell), arising from the epithelial lining. Bladder cancers are broadly classified as noninvasive or invasive (muscle-invasive and metastatic) cancers. The noninvasive and invasive subtypes are thought to arise from distinct biological pathways [2]. About 70 to 80% of newly diagnosed bladder cancers are noninvasive. The initial treatment of noninvasive cancer involves a complete transurethral resection followed by adjuvant intravesical therapy [3]. As many as 70% of noninvasive cancers recur, necessitating life-long surveillance, and up to 25% will progress to more advanced disease [4, 5].

For patients with muscle-invasive, nonmetastatic disease, radical cystectomy with bilateral pelvic lymph node dissection remains the mainstay of treatment. Recurrence can be frequent even after surgery. For example, about 50% of patients with deep, muscle-invasive disease will develop metastatic disease even after surgery [6]. Thus, systemic platinum-based chemotherapy, either in a neoadjuvant or adjuvant setting, is considered a component of the standard care for this disease. Metastatic disease is usually treated with chemotherapy, but the median survival even with the best chemotherapy is often only about 14 months [7].

Improving survival outcomes in advanced bladder cancer will require moving beyond conventional histopathologic evaluation such as stage and grade. Molecular biomarkers have the potential to more accurately determine prognosis and assign patients to appropriate treatments. Such biomarkers are already being used in other solid tumors such as breast, colon, and lung. For example, high expression of the ERCC1 gene is prognostic of improved survival and predictive of reduced response to platinum-based therapy in non-small cell cancer (NSCLC) [8]. Many promising biomarkers are now being evaluated for bladder cancer, offering the potential of improving clinical outcomes. As our understanding of the molecular pathways in bladder cancer improves, more personalized therapies can be delivered that are potentially more active and hopefully less toxic. In this paper, we will review the current chemotherapeutic treatments for advanced disease, highlight the potential role of biomarkers, and remark on the future direction of bladder cancer care.

## 2. Treatment of Muscle-Invasive Bladder Cancer

The current standard treatment in the United States for muscle-invasive bladder cancer is radical cystectomy with bilateral pelvic lymph node dissection. These patients often develop metastatic disease despite aggressive surgical intervention. In organ-confined pT2 disease, the 5-year survival rate is approximately 68% [9]. Patients with more deeply invasive tumors have lower five-year survival rates of 30 to 50% [10]. Relapse is due to the presence of occult micrometastases. 

### 2.1. Neoadjuvant Therapy

The rationale for neoadjuvant chemotherapy prior to cystectomy is to treat micrometastatic disease that is present at diagnosis. It also helps downstage the tumor and increases the potential for complete resection of tumor. Furthermore, neoadjuvant chemotherapy allows delivery of systemic therapy through intact blood vessels and can be better tolerated before the patient is debilitated by surgery. There is level I evidence with two randomized trials to support the use of neoadjuvant chemotherapy [11, 12]. The largest neoadjuvant chemotherapy trial was conducted by the Medical Research Council/European Organisation for Research and Treatment of Cancer. In this phase III trial, 976 patients with high-grade T2-T4a, N0-NX, M0 bladder cancer were randomly assigned to three cycles of neoadjuvant chemotherapy (CMV: cisplatin, methotrexate, and vinblastine, *n* = 491) or no chemotherapy (*n* = 485) then followed by institution's choice of therapy with radical cystectomy and/or radiation therapy [11]. At three years, the pathologic complete response (pCR) in the neoadjuvant group was 33%. Although there was a 5.5% survival benefit at three years (55.5 for chemotherapy versus 50% for no chemotherapy), it did not reach statistical significance. At eight-year followup, results showed a statistically significant 16% reduction in the risk of death, corresponding to an increase in 10-year survival from 30 to 36% after neoadjuvant chemotherapy [13].

A US Intergroup trial (INT 0080) randomized 307 patients with stage T2-4, N0, M0 bladder cancer to three cycles of neoadjuvant methotrexate, vinblastine, doxorubicin, and cisplatin (MVAC) or no chemotherapy followed by cystectomy [12]. The trial took 13 years to accrue. At a median followup of 8.7 years, pCR with MVAC was higher (38 versus 15%). Patients treated with MVAC showed an improvement in median overall survival (77 versus 46 months, *P* = 0.06) and five-year overall survival (57 versus 43%, *P* = 0.06) that were of borderline statistical significance.

The benefit of neoadjuvant chemotherapy was confirmed by a meta-analysis of 11 randomized trials with 3005 patients. It was found that those who received neoadjuvant platinum-based combination chemotherapy compared to local therapy alone had a 14% reduction in the risk of death [14]. This was equivalent to a 5% absolute improvement in overall survival from 45 to 50% at 5 years (*P* = 0.003). There was also a significant disease-free survival benefit (*P* < 0.0001), equivalent to a 9% absolute improvement at 5 years.

Even though CMV and MVAC were used in the above-mentioned prospective trials, the gemcitabine and cisplatin (GC) combination is commonly used in the neoadjuvant setting. There is no randomized trial supporting the use of the GC regimen in this setting. Clinicians mainly extrapolated the data in the metastatic setting showing similar efficacy but better tolerability with the GC regimen compared to MVAC.

Neoadjuvant cisplatin-based chemotherapy is not widely used in practice even though there is level I evidence indicating a significant survival advantage for patients with muscle-invasive bladder cancer [15]. An analysis of 7,161 patients with stage III bladder cancer from the National Cancer Database between 1998 to 2003 revealed that perioperative chemotherapy was administered to 11.6% of patients with stage III bladder transitional cell carcinoma with 10.4% receiving adjuvant chemotherapy and 1.2% receiving neoadjuvant chemotherapy [16]. A more recent analysis from the same database between 2003 and 2007 showed a slight increase in the use of neoadjuvant chemotherapy (up to 13% of patients in 2007) [17]. One major concern is the potential for disease progression due to delayed definitive treatment in patients who do not respond to neoadjuvant chemotherapy. There is a critical need to identify those who will respond favorably to neoadjuvant chemotherapy.

### 2.2. Adjuvant Therapy

The postoperative adjuvant approach has several advantages. It allows for selection of patients at highest risk for surgical failure based on accurate pathologic staging, avoids delay in potentially curative surgery especially in nonresponders to chemotherapy, and prevents overtreating patients who may have a reasonable outcome from surgery alone. The drawbacks include difficulty in administering chemotherapy postoperatively as a result of declines in performance status or development of complications and delays in treating occult metastatic disease.

The results from the adjuvant trials have been conflicting and difficult to interpret. Two small trials have shown a significant difference in favor of adjuvant chemotherapy. One trial randomized 91 patients with pT3-T4a or node-positive bladder cancer to four cycles of adjuvant chemotherapy or to observation after radical cystectomy [18]. The chemotherapy regimen employed was cisplatin, cyclophosphamide, and doxorubicin. There was a significant improvement in median survival in the adjuvant group (4.3 versus 2.4 years, *P* = 0.0062) and percentage free of progression (70 versus 46%, *P* = 0.01) at three years compared to observation. The three-year overall survival in patients was not statistically significant though. A German trial randomized 49 similarly high-risk patients (pT3b, pT4a, and/or positive pelvic lymph nodes) to MVAC, MVEC (methotrexate, vinblastine, epirubicin, and cisplatin), or observation [19]. It was terminated early when an interim analysis showed significant improvement in three-year progression-free survival (PFS) (63% versus 13%, *P* = 0.002). Ten-year survival data from this trial still favored adjuvant chemotherapy over surgery alone [20]. Of note, most patients randomly assigned to observation after cystectomy were not given chemotherapy at the time of relapse.

Other trials revealed no benefit with adjuvant chemotherapy. A Swiss trial randomized 77 patients with muscle-invasive (pT2), nonmetastatic bladder cancer to observation or 3 cycles of cisplatin. There was no significant difference in the overall survival at 5 years between the treatment and control group (57 versus 54%) [21].

The positive trials were criticized for having major deficiencies including small sample size, early stopping of patient entry, inappropriate statistical analyses, and poor reporting of results [22]. A meta-analysis based on 491 patients from six trials reported an absolute improvement in survival of 9% at 3 years [23]. It did acknowledge that the results were drawn from limited data and, therefore, was insufficient to base reliable treatment decisions. Many large cooperative group trials were designed to answer the adjuvant chemotherapy question but have been terminated prematurely due to problems with accrual. A recent large retrospective cohort study consisting of 3,947 patients from 11 centers demonstrated a significant survival benefit with adjuvant chemotherapy in patients at the highest risk of disease progression, such as those with advanced pathologic stage and nodal involvement [24]. In the 20% of patients with the highest risk disease, the median survival was 25 weeks for those receiving adjuvant chemotherapy and 19.2 weeks for those who did not. As of now, adjuvant chemotherapy is commonly used in patients with pT3-T4 or node-positive disease who have not received neoadjuvant chemotherapy.

## 3. Treatment of Metastatic Bladder Cancer

The standard treatment for patients with metastatic bladder cancer is systemic chemotherapy. Although bladder cancer is a chemosensitive tumor, the median survival with chemotherapy is only around 14 months. The five-year survival rate remains poor at about 15% [7]. Cisplatin-based combination therapy is considered first-line based on a prospective randomized trial that compared cisplatin alone to MVAC in 269 patients [25]. Patients treated with MVAC had significant improvement in response rate (39 versus 12%), PFS (10 versus 4.3 months), and overall survival (12.5 versus 8.2 months). Toxicity is a major concern with the MVAC regimen, particularly leukopenia, febrile neutropenia, mucositis, and nausea/vomiting. Only 24% of the patients received full-dose MVAC without dosage modifications. Five patients (4%) in the MVAC group plus 2 patients who were switched over to the MVAC regimen died from treatment-related toxicity.

In the search for a less toxic yet still effective regimen, a randomized phase III trial of 405 patients compared GC to MVAC [26]. The overall response rate (49 versus 46%), time to progression (7.4 versus 7.4 months), and median survival (13.8 versus 14.8 months) were similar for both regimens. Patients treated with GC were more likely to complete treatment with fewer dose adjustments. They had experienced less grade 3 or 4 neutropenia, neutropenic sepsis, and mucositis. An updated analysis showed similar 5-year overall survival rates of 13.0% for GC and 15.3% for MVAC [7]. Although the trial was not designed as an equivalence trial, GC has been adopted by many as the standard first-line treatment based on similar efficacy and lesser toxicity.

Another randomized phase III trial assigned 263 patients to high-dose-intensity MVAC (2-week cycles) with granulocyte colony-stimulating factor (G-CSF) versus standard MVAC (4-week cycles) to see if overall survival can be improved [27]. High-dose-intensity MVAC had significantly improved complete response (21 versus 9%), overall response (62 versus 50%), and the median PFS time (9.1 versus 8.2 months), but there was no difference in overall survival. Toxicity was less with high-dose-intensity MVAC which was attributed to the use of G-CSF. In a subsequent seven-year update, there was a significant survival advantage at 5 years with high-dose-intensity MVAC (21.8 versus 13.5%) [28]. This regimen is becoming more popular because of improved outcomes, shorter duration, and less toxicity compared to standard MVAC.

## 4. Biomarkers for Personalized Chemotherapy

Currently, chemotherapy for bladder cancer is taking the approach of one formula for all. Most patients presently receive a platinum-based regimen, usually GC. However, only about half of the bladder cancers will respond to chemotherapy. Extensive research is ongoing to better understand the biology of the disease process in order to improve clinical outcomes. Conventional prognostic factors such as the grade and stage of the tumor and tools like nomograms are useful in predicting the outcomes associated with surgery and the risk of recurrence but are inadequate in predicting response to chemotherapy [29, 30]. Biomarkers have the potential not only to further identify high-risk bladder cancer patients, but also to help select therapy for those who will benefit most from it. A personalized approach to chemotherapy has the potential to reduce toxicity and improve clinical outcomes (Table 1). 

### 4.1. Single Gene Markers

#### 4.1.1. p53

p53 is the most studied biomarker in bladder cancer and many other cancer types. It plays a critical role in the regulation of cell cycle and is also involved in DNA damage and repair, cell cycle arrest, and apoptosis [31]. Alteration in the p53 leads to a loss of its tumor suppressor function and is thought to be a key event in carcinogenesis. It has been reported that overexpression of p53 in the nucleus, as detected by immunohistochemistry, was associated with increased risk of recurrence and death in bladder cancer [32, 33]. However, a meta-analysis of 117 studies comprising of 10,026 patients showed that changes in p53 were only weakly predictive of recurrence, progression, and mortality in bladder cancer [34]. p53 overexpression was predictive of recurrence, progression, and mortality in 27%, 50%, and 29% of eligible studies, respectively.

In addition to having prognostic qualities, studies have suggested that p53 may be predictive of benefit to chemotherapy [35]. A retrospective analysis of patients treated with adjuvant therapy found that patients with p53 alteration had increased sensitivity to treatment and had more benefit from adjuvant chemotherapy [36]. Since p53 is involved in cell cycle arrest and DNA repair, the lack of a normal p53 could result in greater cancer cell killing when exposed to DNA-damaging therapy. This hypothesis was put to the test in a phase III trial that focused on patients with pT1 or pT2, N0, M0 bladder cancer who had undergone a radical cystectomy and bilateral pelvic lymphadenectomy [37]. This group is not usually treated with adjuvant therapy but has a recurrence rate of about 30%. Those whose tumors demonstrated ≥ 10% nuclear immunoreactivity for p53 were randomized to three cycles of adjuvant MVAC versus observation while p53-negative patients were observed. The trial tried to confirm p53 as a predictive biomarker and to see whether p53-altered tumors would respond better to MVAC. A total of 521 patients were registered, 499 underwent p53 assessment, 272 (55%) were positive, and 114 (42%) were randomly assigned. Unfortunately, due to the high patient refusal rate, lower than expected event rate, and failures to receive assigned therapy, accrual was halted and questions about p53 as a biomarker remain.

#### 4.1.2. ERCC1

Nucleotide excision repair pathway plays a major role in DNA damage repair, and the excision repair cross-complementing group 1 (ERCC1) is key member [38]. The cytotoxic effect of cisplatin is attributed to the formation of bulky DNA adducts. ERCC1 helps remove these adducts and thus may cause resistance to platinum agents [39]. In NSCLC, high ERCC1 is associated with an improved prognosis and predictive of reduced response to platinum-based therapy [40].

Studies have also been done to evaluate ERCC1 as a biomarker in patients with advanced bladder cancer treated with cisplatin-based chemotherapy. Bellmunt et al. performed gene expression analysis by using real-time quantitative PCR in tumors of 57 patients with metastatic or locally advanced, surgically incurable patients who were treated with either GC or GC plus paclitaxel [41]. At a median followup of 19 months, the median survival was significantly longer in patients with low ERCC1 level (25.4 versus 15.4 months, *P* = 0.03). Low ERCC1 level is predictive of the progression-free survival in patients who received adjuvant cisplatin-based chemotherapy. At 5 years, only 45% of patients with low ERCC1 level had progressed versus 70% of patients with high ERCC1 level (hazard ratio = 0.52, *P* = 0.03) [42]. There was also a PFS advantage found in metastatic patients with low ERCC1 (10.6 versus 8.4 months, *P* = 0.03) [43]. However, prospective randomized controlled clinical trials are needed to determine the true value of ERCC1 expression in predicting response to platinum-based chemotherapy.

#### 4.1.3. RRM1

Ribonucleotide reductase subunit M1 (RRM1) gene encodes one of two nonidentical subunits of ribonucleotide reductase, an essential enzyme involved in the production of deoxyribonucleotides for DNA synthesis and repair [44]. Another function of RRM1 is suppression of cell migration and metastasis formation [45]. It is the molecular target of gemcitabine, an antimetabolite used in several malignancies including lung and bladder. Increased expression of RRM1 is associated with increased survival of patients with resected NSCLC [46]. In patients with early stage NSCLC who had only received surgical treatment, the overall survival for those with high RRM1 expression was more than 120 months compared to 60.2 months for those with low RRM1 (*P* = 0.02) [47]. The survival advantage was limited to patients with tumors that also expressed ERCC1. On the other hand, high RRM1 expression appears to be a predictor of decreased response to gemcitabine/platinum chemotherapy [48].

RRM1 may have utility as a biomarker in bladder cancer as well. In the Bellmunt study where patients received GC with or without paclitaxel, there was a trend towards longer time in progression in tumors with low RRM1 expression [41]. High RRM1 expression was found to be prognostic for improved survival in younger patients (aged < 70 years) with muscle-invasive bladder [49]. The median overall survival was 10.6 years in younger patients high RRM1 expression versus 1.6 years in older patients (*P* = 0.001), but made no significant difference for patients with low RRM1 expression (2.3 versus 1.6 years in younger and older patients, resp.).

#### 4.1.4. hENT1

Human equilibrative nucleoside transporter 1 (hENT1) is the major molecule of nucleoside transporter proteins. Gemcitabine is a pyrimidine nucleoside analogue that requires plasma membrane nucleoside transporter proteins to enter the cell and exert cytotoxicity. Studies in cultured cells showed that hENT1 deficiency is associated with gemcitabine resistance [50]. The ability for hENT1 to predict benefit in patients receiving gemcitabine has been studied in other cancers such as pancreas and lung [51–53].

In a small study of 12 patients, hENT1 was detected in 3 patients and two of them presented with a complete response to gemcitabine [54]. The mean value of hENT1 was significantly higher in the patients who had a pathological complete response. A larger study evaluated 40 patients with metastatic bladder cancer who received GC-based chemotherapy [55]. Immunohistochemistry on tumor tissue was performed with specific hENT1 antibodies. Eighteen (90%) out of 20 patients with high hENT1 expression showed a response to chemotherapy whereas only 7 (35%) of 20 patients with low hENT1 expression responded. A significantly longer median survival was seen in patients with high hENT1 expression than those with lower levels (17.3 versus 11.6 months, *P* = 0.003). Therefore, hENT1 might be a relevant biomarker in metastatic bladder cancer patients receiving GC-based chemotherapy.

#### 4.1.5. BRCA1

The breast cancer susceptibility gene 1 (BRCA1) is a tumor suppressor gene that is central in DNA repair pathways. It encodes a nuclear protein that functions in multiple biological processes, including gene transcription, DNA damage repair, and apoptosis [56]. Low expression of BRCA1 has been found to increase sensitivity to cisplatin-based chemotherapy in ovarian cancer and NSCLC [57, 58]. A similar result was found in bladder cancer where a significant pathologic response to neoadjuvant cisplatin-based chemotherapy was attained in 66% of patients with low/intermediate BRCA1 levels compared with 22% of patients with high BRCA1 levels (*P* = 0.01) [59]. Median survival was prolonged in patients with low/intermediate compared to high BRCA1 levels (168 versus 34 months, *P* = 0.002). BRCA1 may be a useful tool in the selection of patients for neoadjuvant cisplatin-based chemotherapy.

#### 4.1.6. MDR1

The multidrug resistance gene 1 (MDR1) encodes P-glycoprotein (Pgp), a membrane protein that acts as an energy-dependent cellular efflux pump. Pgp can reduce intracellular concentrations of chemotherapy drugs like anthracyclines and vinca alkaloids which are components of MVAC, resulting in decreased cytotoxicity. Furthermore, it appears that chemotherapy drugs induce MDR1 and lead to drug resistance [60]. In patients with locally advanced bladder cancer receiving adjuvant chemotherapy, high MDR1 expression is associated with inferior survival [42]. After 2 years, more than 65% of patients with high MDR1 expression had progressed compared to only 25% of patients with low MDR1 expression. After 5 years, only 23% of patients with high MDR1 expression were still alive versus 62% of patients with low MDR1 expression (HR 0.25, *P* = 0.0006).

#### 4.1.7. Bcl-2

B-cell lymphoma 2 (Bcl-2) is the representing member of the large Bcl-2 family that is important in regulating cellular apoptosis. It was originally identified in follicular lymphoma at the site of the t(14; 18) translocation [61]. Bc1-2 is an antiapoptotic protein that is localized in intracellular membranes and has been found to have prognostic value in bladder cancer. Overexpression of Bcl-2 is associated with reduced survival in patients with invasive bladder cancer and lower response rate to chemotherapy [62]. In patients with invasive disease treated with radiotherapy only, Bcl-2 positivity was found to be related to poor local control (36 versus 72%) as well as to shorter disease-specific survival (74 versus 94%) at 3 years [63]. In a study of four apoptosis markers, including Bcl-2, caspase-3, p53, and survivin, in patients treated by radical cystectomy, Bcl-2 was independently associated with higher pathologic stage, probability of disease recurrence (HR 2.24, *P* < 0.001), and disease-specific mortality (HR 2.06, *P* = 0.001) [64].

#### 4.1.8. MicroRNA

MicroRNA (miRNA) is small noncoding regulatory RNA molecules with the stem-loop secondary structure. Its size ranges from 17 to 25 nucleotides. It was first found in worms but later was found in most eukaryotic cells [65, 66]. It works as a posttranscriptional regulator of genes by binding to the complementary 3′ untranslated region of target mRNA and degrading the target mRNA or suppressing translation. Because one miRNA can bind to and regulate the expression of multiple mRNAs, it works as a master posttranscriptional regulator of gene expression. miRNAs are involved in almost every aspects of oncogenesis. They have been found to be upregulated or downregulated depending on their corresponding functions as a tumor suppressor or promoter [67]. Multiple miRNAs have been found to be involved in bladder cancer [68]. Genome-wide deep sequencing of clinical specimens revealed that a set of miRNAs was aberrantly expressed in bladder cancer when compared to the normal matched control [69]. Many of the miRNA dysregulated in bladder cancer were repeatedly identified in other cancer types, but some of them have not been reported before [70]. Some of these miRNAs affect several signaling and metabolic pathways that can be potentially targeted for cancer therapy in bladder cancer. Among those miRNAs, MiR-34a is frequently downregulated or deleted in several cancer types [71, 72]. It is a known downstream effector of p53 that regulates several components of the p53 pathway such as Cdk6 and E2F3. Researchers at University of California at Davis found that transfection of bladder cancer cell lines with pre-miR-34a followed by cisplatin treatment results in a dramatic reduction in clonogenic potential and induction of senescence compared to treatment with cisplatin alone. Analysis of 27 preneoadjuvant chemotherapy patient samples revealed many of the patients who subsequently did not respond to treatment (based on surgical resection postchemotherapy and 5-year survival data) express lower levels of miR-34a [73]. However, the clinical significance needs to be defined in prospective trials.

### 4.2. Combination of Genetic Markers

Individual gene biomarkers may not adequately capture the complex molecular activities in tumor cells. To accurately predict chemosensitivity requires a large body of information. For example, there are over 150 genes involved in the major DNA repair pathways that are relevant to platinum-DNA adducts alone. Several studies have been conducted using a combination of genetic markers to predict response to chemotherapy. Takata et al. analyzed the gene expression profile of 27,648 genes from 27 invasive bladder cancers using a cDNA microarray [74]. Profiles of tumors from patients who responded to MVAC neoadjuvant chemotherapy were compared to nonresponders to develop a scoring system of 14 predictive genes. This system was able to accurately predict drug response in 8 out of 9 patients. It was applied to 22 additional cases of bladder cancer and correctly predicted clinical response for 19 cases [75]. Another approach uses the coexpression extrapolation (COXEN) algorithm derived from expression microarray data of the National Cancer Institute (NCI)-60 cell line panel to predict drug sensitivity of bladder cancer cell lines [76]. The COXEN-based gene expression model was able to effectively stratify chemosensitivity and predict the 3-year overall survival in patients treated with MVAC [77].

### 4.3. DNA Damage as a Predictor of Chemoresistance

The major limitation of studying individual genes or gene combination in cancer specimens is that *in vivo* patient pathophysiological changes cannot be analyzed. Furthermore, genetic alterations can be so complicated that they cannot be fully explored. For example, over 700 genes are involved in cellular response to platinum chemotherapy [78]. Even though all these genes can be studied with the currently available high throughput analysis, such as microarray and whole genome or transcriptome sequencing, the *in vivo* factors (such as drug metabolism, distribution, local vasculature, and drug delivery) and external factors (such as dose calculation and administration) cannot be analyzed.

**Figure 1:**
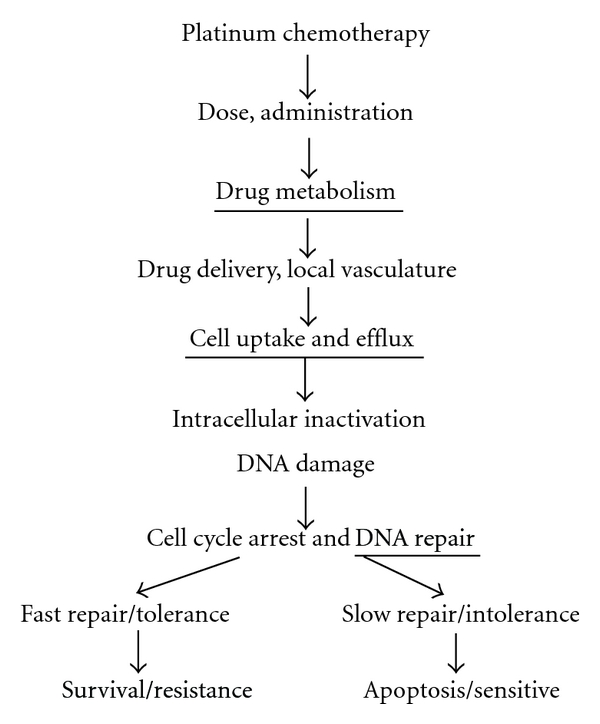
Pathways leading to chemotherapy-induced cell death and resistance. The major steps are shown in the sequential order. DNA damage is the critical step in this response pathway. Cells with low chemotherapy-induced DNA damage will survive chemotherapy and are chemoresistant. We propose that chemoresistance can possibly be identified by measuring chemotherapy-induced DNA damage and that some of the underlying resistance mechanisms can potentially be elucidated by measuring the other major steps such as metabolism, cell uptake/efflux, and DNA repair. The underlined steps can be determined with AMS.

To overcome these limitations, we have taken a radically different approach to study chemoresistance to platinum therapy *in vivo* under the physiological conditions [79, 80]. As alkylating agents, platinum analogs kill cancer cells mainly through induction of DNA damage (adducts). We hypothesize that cancer cells with low platinum-induced DNA can survive chemotherapy and are platinum resistant (Figure 1). Therefore, measurement of platinum-induced DNA damage may allow for identification of chemoresistance. By analyzing some other major steps along the chemotherapy pathway, such as drug metabolism and DNA repair, some of the major underlying chemoresistant mechanisms can be determined that help design of personalized chemotherapy to overcome resistance. For example, if the chemoresistance to platinum chemotherapy is secondary to increased DNA repair (as measured by fast decrease of DNA adducts over time), platinum drugs can be combined with a DNA repair inhibitor, such as a PARP (Poly ADP ribose polymerase) inhibitor, to overcome resistance. Several studies already showed that low DNA damage induced by alkylating agents correlated with chemoresistance with a few exceptions [81–90]. However, the major limitation for those studies to be in clinical application is that the technologies used in those studies are not sensitive enough for clinical applications because patients had to receive toxic therapeutic to high-dose chemotherapy before DNA damage could be detected. 

We have developed a highly sensitive technology to measure carboplatin or oxaliplatin-induced DNA damage using accelerator mass spectrometry (AMS). It is an isotope ratio mass spectrometric method that precisely determines the concentration of very rare (<1 : 10^9^) isotope in an isolated sample [91, 92]. Compared with the other technologies, the advantage of using AMS to detect DNA adducts/damage is its high sensitivity and precision. It can detect ^14^C at the zeptomole (zmole, 10^−21^ mole) level per mg of carbon with the precision as low as 0.25% [93]. To measure platinum-induced DNA damage, ^14^C-labeled platinum analogs are used. When DNA damage is induced, platinum analogs together with ^14^C are conjugated to genomic DNA. By measuring the amount of ^14^C on genomic DNA, the amount of DNA damage can be calculated. Because of the supersensitivity of AMS, this approach allows the measurement of DNA adducts without exposing cancer cells or patients to toxic chemotherapy. To perform this test, cancer cells/patients are first treated with one nontoxic microdose (1% of therapeutic dose or less) of ^14^C-labeled platinum drug before cancer patients undergo biopsy or surgical resection. Biopsy is required for most cancer patients before chemotherapy can be administered as the standard of care. Some of the tumor specimens will be used for the AMS analysis. Only a few milligrams of tumor tissue are needed for detection of DNA adducts with AMS. One major limitation of this approach is that cisplatin cannot be studied as there is no carbon atom in cisplatin molecule to allow for ^14^C labeling. Fortunately, the chemoresistance spectrum of cisplatin and carboplatin is very similar as these two drugs form the same platinum-DNA cross-links [94].

We have performed extensive preclinical studies to show the feasibility of this microdosing approach [79, 80, 95]. Microdosing with ^14^C-carboplatin could induce DNA damage, the physiological target of therapeutic carboplatin. The DNA damage induced by microdosing is highly linearly proportional to that of therapeutic carboplatin (*P* < 10^−15^), suggesting that the microdosing approach can be used to predict DNA damage induced by therapeutic dose of carboplatin. In studies with cell lines, the level of DNA damage is superior in predicting chemoresistance when compared to the ERCC1 level [80]. This finding is consistent with the underlying pathophysiology of resistance to platinum in that ERCC1 is one of many proteins involved in DNA repair while DNA damage levels are the cumulative results of formation and repair of DNA damage. Because carboplatin is rarely used in clinic as a single agent, we determined and found that addition of gemcitabine had little effects on the formation of platinum-DNA adducts in a bladder cancer cell line [95]. Based on these preclinical studies, a Phase 0 microdosing trial has been designed and is currently going on to determine if the DNA damage induced by the microdosing ^14^C-carboplatin correlate with chemotherapy outcomes (http://clinicaltrials.gov/ identifier no. NCT01261299).

## 5. Conclusion

Cancer therapeutics is moving towards personalized medicine to select the most effective therapy while avoiding the ineffective and/or toxic therapy based on the underlying pathophysiology of the patients and their tumors. In bladder cancer, questions remain about who will benefit most from chemotherapy as only around 50% of bladder cancer patients will have tumor response from chemotherapy. Biomarkers have shown promise in prognostication and in selecting therapy and might help answer some of these questions. For example, ERCC1 status may be important to be determined before giving platinum-based chemotherapy, or hENT1 or RRM1 before gemcitabine. Combinations of biomarkers (including microRNA) can offer more accuracy in prediction than individual ones. Our group is using a microdosing approach to identify resistance to platinum drugs [79, 80]. The efficacy needs to be determined in large clinical trials. A better understanding of tumor biology and pathogenic pathways will hopefully lead to more molecularly targeted therapy. Research is ongoing to test agents that target the fibroblast, epidermal, and vascular endothelial growth factor pathways [96]. The hope is that in the future targeted therapy will augment cytotoxic chemotherapy and that biomarkers will be able to risk stratify patients and help optimize therapy.

## Figures and Tables

**Table 1:** Single gene markers for prognosis and prediction of response in bladder cancer.

Markers	Function	Relation to bladder cancer
p53^34^	Tumor suppressor, DNA repair, and apoptosis	p53 mutation associated with high recurrence and progression
ERCC1^41-43^	DNA repair	Low expression associated with increased response to platinum-based chemotherapy
RRM1^41, 49^	Synthesis of deoxyribonucleotides	High expression with improved survival and possibly resistance to gemcitabine
hENT1^54-55^	Nucleoside transporter	Sensitivity to gemcitabine
BRCA1^59^	DNA repair	Low expression related to increased response and prolonged survival
MDR1^42, 60^	P-glycoprotein efflux pump	High expression associated with resistance to chemotherapy
Bcl-2^64^	Antiapoptosis protein	Associated with more advanced stage and worse prognosis
